# Utility of cerebrospinal fluid multiplex polymerase chain reaction assay in children suspected of meningitis or encephalitis in a tertiary care hospital in Thailand

**DOI:** 10.1016/j.ijregi.2026.100927

**Published:** 2026-05-30

**Authors:** Piyarat Suntarattiwong, Prangtip Khunsri, Chompunut Na Nakorn, Sirorat Suwannachote, Busayapan Jaipean, Tawee Chotpitayasunondh

**Affiliations:** 1 Department of Pediatrics, Queen Sirikit National Institute of Child Health, Bangkok, Thailand; 2 College of of Medicine, Rangsit University, Bangkok, Thailand; 3 Department of Pathology, Queen Sirikit National Institute of Child Health, Bangkok, Thailand

**Keywords:** Meningitis Encephalitis Panel, Encephalitis in children, Encephalitis in tropical countries

## Abstract

**Background:**

The BioFire®FilmArray® meningitis/encephalitis panel (ME panel)identifies 14 cerebrospinal fluid (CSF) pathogens. It has not been studied in Thailand,where the epidemiology of central nervous system (CNS) infection may differ from thatof countries with temperate climates. We conducted a study to evaluate theperformance of the ME panel in Thai children.

**Method:**

We conducted a prospective study in children suspected of meningitis orencephalitis at the Queen Sirikit National Institute of Child Health in Bangkok, Thailand.In addition to routine tests, CSF was tested simultaneously with an ME panel.

**Results:**

Seventy-three children diagnosed with meningitis or encephalitis wereenrolled. Of them, 47 (64.4%) had an infectious etiology, 21 (28.8%) a non-infectiousetiology (mainly immune encephalitis), and 5 had an undetermined etiology. Thirtypatients had pathogens confirmed: 11 bacteria, 5 *Mycobacterium tuberculosis*, 5Arboviruses (Dengue and Chikungunya), 8 herpesviruses, and 2 influenza viruses.One patient had both Chikungunya and Influenza virus. Of them, 13 pathogens (43%)were not covered by the ME panel, including Klebsiella spp., Acinetobacter spp., *M. tuberculosis*, Dengue, Chikungunya, and Influenza virus. The ME panel provided 59% sensitivity and 95% specificity when excluding these pathogens.

**Conclusion:**

The ME panel is a reliable tool for supporting the management of CNSinfections due to its high specificity and rapid turnaround time. However, the diversity ofpathogens that cause CNS diseases poses a challenge to its use in tropical countries.

## Highlights

• The FilmArray® meningitis/encephalitis panel rapidly detects multiple pathogens in cerebrospinal fluid simultaneously.
• Its specificity is high; sensitivity is low due to some pathogens in the tropics not being included.
• Using the meningitis/encephalitis panel, clinicians should be aware of the clinical manifestations and epidemiology of central nervous system diseases in tropical countries.

## Graphical Abstract

**Figure:**
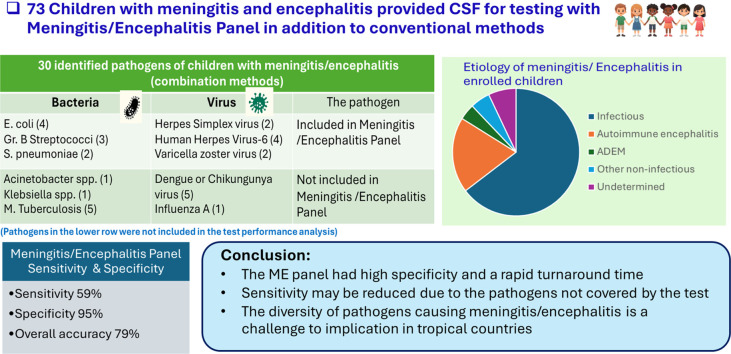

## Introduction

Meningitis and encephalitis are life-threatening central nervous system (CNS) diseases that can be caused by infectious or non-infectious etiologies. Cerebrospinal fluid (CSF) analysis is critical for determining the etiology of meningitis and encephalitis. Standard CSF testing includes a white blood cell count with differential, glucose, and protein, which can help differentiate the causes of meningitis and encephalitis in conjunction with clinical features and neuroimaging. Initial results are typically returned within hours or even faster, but they are not specific. Conventional tests include Gram stain, Latex agglutination, bacterial culture, and nucleic acid amplification tests, which provide a more specific etiologic diagnosis. Their limitations, however, are either low sensitivity (e.g., Gram stain) or the need for longer turnaround times (e.g. culture). Latex agglutination and nucleic acid amplification tests enable faster turnaround times. Latex agglutination showed lower sensitivity than the latter, but both are uniformly designed to target specific pathogens and will miss non-target pathogens [1].

In recent years, rapid multiplex molecular tests have been developed to identify potential CNS infectious pathogens in CSF, thereby facilitating prompt antimicrobial administration. The benefits of these tests include generally high sensitivity and specificity, reduced turnaround time by combining multiple common pathogens in a single test, and a lower CSF volume required, which collectively improve the diagnosis of meningitis and encephalitis, particularly in children [[2], [3], [4]].

The BioFire®FilmArray® meningitis/encephalitis (ME) panel is a qualitative multiplex polymerase chain reaction (PCR) assay used on the FilmArray system. It identifies up to 14 potential pathogens, including 6 bacterial [*Escherichia coli* K1, *Haemophilus influenzae, Listeria monocytogenes, Neisseria meningitidis, Streptococcus agalactiae*, and *Streptococcus pneumoniae*], 7 viral [Cytomegalovirus (CMV), Enterovirus (EV), Herpes simplex virus 1 (HSV-1), Herpes simplex virus 2 (HSV-2), Human herpesvirus 6 (HHV-6), Human parechovirus (HPeV), and varicella-zoster virus (VZV)], and 1 fungal pathogen, *Cryptococcus neoformans/gattii* with less than 0.5 mL of CSF [5].

The ME panel has been available since 2015, with published data demonstrating its usefulness in reducing antimicrobial use, shortening hospitalization length of stay, and lowering hospital costs [[6], [7], [8]]. Nevertheless, most studies have been conducted in temperate-climate countries, where the epidemiology of meningitis and encephalitis may differ from that of CNS infections in tropical countries. Therefore, we conducted a study to evaluate the performance of the ME panel in children in a tertiary care hospital in Thailand.

## Materials and methods

We conducted a prospective study at Queen Sirikit National Institute of Child Health, a large public hospital and referral center in Bangkok, Thailand, from July 1, 2020, to December 31, 2023. The enrollment criteria were: (1) age between 1 day and 15 years, (2) admission to the hospital with suspected meningitis or encephalitis, and (3) consent to provide at least 0.5 mL of CSF for analysis using the ME panel, in addition to the conventional CSF analysis. Children with an alternative diagnosis, for example, a brain tumor, and those with an intraventricular catheter, were excluded.

The enrolled children underwent routine laboratory investigations, including CSF white blood cell count with differential, glucose, and protein levels. Standard microbiological tests, such as Gram stain, bacterial culture, and latex agglutination, were routinely performed at our hospital. Additional CSF analyses, such as PCR for herpes simplex virus, tuberculosis, and autoimmune markers, were performed on a case-by-case basis, depending on the patient’s clinical information.

In addition, the CSF was analyzed using the ME panel. The procedure followed the FilmArray ME Panel Instruction Booklet [9]. In brief, the system employs a freeze-dried reagent pouch that stores components necessary for nucleic acid extraction and PCR reactions. The user injects hydration solution and a sample/buffer mixture prepared with 200 µL of CSF specimen into the pouch using the FilmArray loading station. Nucleic acid extraction, purification, and multiplexed nested PCR are performed in the enclosed pouch using the FilmArray instrument to simultaneously test for the 14 pathogens described previously. All processes were performed immediately by the lab technician upon receipt of the CSF samples, and the results were reported to the attending physician upon completion of the procedure.

The diagnostic performance of the ME panel was compared with the final diagnosis of meningitis and encephalitis, as determined by hospital discharge records and consultations with pediatric infectious disease and neurology specialists. STATA 16.1 (Stata Corp, Texas, USA) was used for statistical analysis.

## Results

Of 118 children enrolled, 73 were diagnosed with meningitis, encephalitis, or meningoencephalitis. The median age was 11.7 months (range, 2.9-81.3 months). Most enrollment (76.7%) occurred within the first 3 days of illness. The clinical diagnosis included 24 (32.9%) cases of meningitis, 42 (57.5%) encephalitis, and 7 (9.6%) meningoencephalitis. Among them, 47 (64.4%) had infectious origins, 21 (28.8%) were non-infectious, and 5 (6.8%) had undetermined causes. Non-infectious included 16 immune-mediated CNS disorders and 4 other causes, which were, Kawasaki disease (2), neuropsychiatric SLE (1), and subacute sclerosing panencephalitis (1) (Table 1).

**Table 1:** Patients’ characteristics.

Characteristics	Number (%) (N = 73)
Median age in months (IQR)	11.7 (2.91, 81.3)
Day of illness at enrollment (%)	
1-3 days	56 (76.7)
4-7 days	6 (8.2)
>7 days	11 (15.1)
Diagnosis (%)	
Meningitis	24 (32.9)
Encephalitis/encephalopathy	42 (57.5)
Meningoencephalitis	7 (9.6)
Etiology (%)	
Infectious	47 (64.4)
Non-infectious	21 (28.8)
- Autoimmune encephalitis	14 (66.7)
- ADEM	3 (14.3)
- Others	4 (19.0)
Undetermined	5 (6.8)

Abbreviations: ADEM, acute disseminated encephalomyelitis; IQR, interquartile range.

Thirty out of 47 children with infectious meningitis and encephalitis (63.8%) had confirmed pathogens. The pathogens were 11 bacteria, 5 *Mycobacterium tuberculosis*, 5 arboviruses, 8 herpes viruses, and 2 influenza viruses. One patient had both Chikungunya and influenza virus documented. The pathogens, diagnostic methods, and ME panel test results are shown in Table 2.

**Table 2:** Pathogens, methods of identification, and ME panel test results.

Pathogen	Clinical diagnosis	Pathogen detected by	ME panel	CSF culture
*E. coli*	Meningitis	Subdural fluid culture	Neg	No growth
*E. coli*^b^	Meningitis	Blood culture	Neg	No growth
*E. coli*	Meningitis	CSF ME panel and culture	Pos	Pos
*E. coli*	Meningitis	CSF ME panel and culture	Pos	Pos
*S. agalactiae*^b^	Meningitis	Blood culture	Neg	No growth
*S. agalactiae*	Meningitis	CSF ME panel and culture	Pos	Pos
Gr. B Streptococcus b	Meningitis	CSF Latex agglutination	Neg	No growth
*S. pneumoniae*^b^	Meningitis	CSF culture (from the first hospital)	Neg	No growth
*S. pneumoniae*^a^	Meningitis	Blood culture CSF ME panel	Pos	No growth
Acinetobacter spp.	Meningitis	Blood culture	N.I.	No growth
Klebsiella spp.	Meningitis	Blood culture	N.I.	No growth
*M. tuberculosis*	Meningoencephalitis	Brain tissue pathology	N.I.	No growth
*M. tuberculosis*	Meningoencephalitis	CSF PCR for TB	N.I.	No growth
*M. tuberculosis*	Meningoencephalitis	Gastric wash PCR for TB	N.I.	No growth
*M. tuberculosis*	Meningoencephalitis	CSF Gene Xpert for TB	N.I.	No growth
*M. tuberculosis*	Meningoencephalitis	CSF profiles and radiology	N.I.	No growth
Dengue virus	Encephalopathy	Blood NS1	N.I.	No growth
Chikungunya virus	Encephalopathy	Blood IgM	N.I.	No growth
Chikungunya virus AND Influenza B	Encephalitis	Blood IgM Nasal swab Influenza Antigen test	N.I.	No growth
Chikungunya virus	Encephalopathy	Blood IgM	N.I.	Neg
Chikungunya virus	Encephalitis	Blood IgM	N.I.	Neg
Influenza A	Encephalopathy	Nasal swab Influenza PCR	N.I.	Neg
HSV-2^d^	Encephalitis	CSF ME panel	Pos	Neg
HSV-2^c^	Encephalitis	CSF PCR (from the referred hospital)	Neg	Neg
HHV-6	Encephalopathy	CSF ME panel	Pos	Neg
HHV-6	Encephalopathy	CSF ME panel	Pos	Neg
HHV-6	Encephalopathy	CSF ME panel	Pos	Neg
HHV-6	Encephalopathy	CSF ME panel	Pos	Neg
Varicella-zoster virus	Encephalitis	Vesicle fluid PCR	Neg	Neg
Varicella-zoster virus	Encephalitis	CSF ME panel	Pos	Neg

Abbreviations: CSF, cerebrospinal fluid; HHV-6, human herpes virus 6; HSV-2, herpes simplex virus type 2; IgM, immunoglobulin M; ME, meningitis/encephalitis; N.I., not included in the ME panel; Neg, negative; PCR, polymerase chain reaction; Pos, positive; TB, tuberculosis.

a CSF collected 20 hours after antimicrobial treatment.

b CSF collected 4 days after antimicrobial treatment.

c CSF collected more than 14 days after antimicrobial treatment.

d CSF conventional PCR sent 10 days after antimicrobial treatment.

The turnaround time for the ME panel was 2 hours. For bacterial etiology, the ME panel detected 4 of 11 cases. Our series included 5 cases of tuberculous meningoencephalitis and 5 cases of Dengue/Chikungunya virus encephalopathy/encephalitis; the ME panel did not test for these pathogens. For herpes viruses, the ME panel detected all cases except one varicella-zoster virus and one herpes simplex virus type 2, as the test were performed more than 14 days after acyclovir administration (Table 2).

To evaluate the diagnostic performance of the ME panel, we calculated its sensitivity, specificity, and accuracy using cases with a conclusive etiology, excluding cases with pathogens that were not covered by the ME panel (13 cases of Acinetobacter spp., Klebsiella spp., *M. tuberculosis*, Dengue, Chikungunya, and influenza virus). A total of 38 cases with either documented pathogens or documented non-infectious ME were included. The results are presented in Table 3, which shows the test sensitivity, specificity, and accuracy of 58.8%, 95.2%, and 78.9%, respectively. However, when the 13 cases were included, the total number was 51, and test sensitivity and accuracy were lower, to 33.3% and 58.8%, respectively.

**Table 3:** Performance of the ME panel.

CSF ME panel	Combined methods, including blood/ other sites specimen PCR and culture		Total
	Positive	Negative	
Positive	10	1	11
Negative	7	20	27
**Total**	17	21	38

Abbreviations: CSF, cerebrospinal fluid; ME, meningitis/encephalitis; PCR, polymerase chain reaction.

Five of the seven false negatives were cases in which CSF was collected 3 days or more after administration of an intravenous antimicrobial agent, one case was *E. coli* meningitis with subdural empyema, in which the bacteria were isolated from subdural fluid only, and one case was varicella-zoster encephalitis, in which the virus was detected from the vesicle fluid PCR (Table 2). There was a false positive case, which was due to a positive *Cryptococcus neoformans/gattii* in a child diagnosed with autoimmune encephalitis. The CSF was then sent for Indian Ink preparation, Cryptococcal antigen, and Cryptococcal culture, all of which were negative.

## Discussion

In our study, meningitis and encephalitis in children were caused by various etiologies; infections accounted for approximately two-thirds of cases, followed by immune-mediated encephalitis, which accounted for nearly one-third. This proportion is similar to other studies in children [10,11]. High-dose intravenous corticosteroids are the first-line therapy for most autoimmune causes of meningitis and encephalitis. At the same time, this treatment may adversely affect patients with a CNS infection. Our study demonstrated the specificity of the ME panel in children with ME syndromes and its utility in supporting neurologists' decisions to initiate corticosteroids or other immunotherapies to improve outcomes in these children. Although *C. neoformans/gattii* false positive occurred in one case of immune-mediated encephalitis, it failed to correlate with the patient’s clinical features. The Cryptococcal antigen test was negative, and the patient continued to receive immunotherapy with favorable treatment outcomes.

The etiologies of meningitis and encephalitis in our patients were predominantly infectious, and approximately two-thirds of the causative pathogens were identified. Implementing the ME panel enhanced the detection of common bacterial pathogens, including *E. coli, S. agalactiae, and S. pneumoniae*. We observed one case of *S. pneumoniae* meningitis that tested positive by the ME panel after 20 hours of intravenous antibiotics. However, both conventional bacterial culture and the ME panel were negative in those who had received antimicrobial therapy for 3 days or more.

Approximately 40% of documented pathogens in our series were *M. tuberculosis*, arboviruses, such as Dengue and Chikungunya viruses, and bacteria not targeted by the ME panel. At the same time, herpes viruses accounted for almost one-third of cases. The positive diagnosis of HHV-6 infection in our patients led to discontinuation of intravenous antibiotics and a reduction in hospital stay. These findings support the use of newer molecular diagnostic tools on a case-by-case basis, with attention to clinical details.

Regarding diagnostic performance, overall agreement was high (79%), with test specificity of 95%. However, when all-cause meningitis and encephalitis in this study were included, its sensitivity dropped from 59% to 33%. Reflecting the pathogens causing CNS infections in Thailand that differ from those in temperate regions. Clinicians should understand the advantages and disadvantages of diagnostic tools to ensure their proper use.

This study has some limitations. First, some enrolled children received empirical antibiotics before CSF was obtained; second, a high proportion were diagnosed with pathogens not covered by the ME panel, resulting in a small number of cases. In addition, our study setting did not have singleplex PCR to confirm HHV-6; the diagnosis was made based on clinical assessment and specialist consensus. These affected the accuracy of the test performance evaluation. However, this is the first report of using ME panel in children in a tropical country like Thailand. We recognized the diagnostic challenges posed by devastating syndromes such as meningitis and encephalitis in children and demonstrated the use of the ME panel as a modern diagnostic tool.

In conclusion, the FilmArray ME panel is a helpful tool for rapid diagnosis of meningitis and encephalitis. It provides rapid microbiological information and supports neurologists in managing patients with immune-mediated encephalitis. Nevertheless, certain important pathogens in the region may not be detected, underscoring the need for clinicians to be aware of the clinical manifestations and epidemiology of CNS diseases in tropical countries. Furthermore, a test panel to cover relevant pathogens in tropical areas should be developed.

## Funding

The study was funded by the bioMérieux BioFire Investigator-Initiated Study Program, BioFire Diagnostics, LLC, and the bioMérieux Asia Pacific team has reviewed the manuscript.

## Ethical approval

This study has been approved by the Research Ethics Review Committee of Queen Sirikit National Institute of Child Health, Document no. 63-050, Approval no. 114/2020.

## CRediT authorship contribution statement

**Piyarat Suntarattiwong:** Conceptualization, Data curation, Methodology, Project administration, Supervision, Writing – original draft, Writing – review & editing. **Prangtip Khunsri:** Data curation, Formal analysis, Investigation, Writing – review & editing. **Chompunut Na Nakorn:** Data curation, Formal analysis, Writing – review & editing. **Sirorat Suwannachote:** Data curation, Investigation, Resources, Writing – review & editing. **Busayapan Jaipean:** Data curation, Investigation. **Tawee Chotpitayasunondh:** Conceptualization, Funding acquisition, Supervision, Writing – review & editing.

## Declaration of competing interest

The authors declare that they have no known competing financial interests or personal relationships that could have appeared to influence the work reported in this paper.
